# The Influence of Functional Flywheel Resistance Training on Movement Variability and Movement Velocity in Elite Rugby Players

**DOI:** 10.3389/fpsyg.2020.01205

**Published:** 2020-06-30

**Authors:** Bruno Fernández-Valdés, Jaime Sampaio, Juliana Exel, Jacob González, Julio Tous-Fajardo, Ben Jones, Gerard Moras

**Affiliations:** ^1^National Institute of Physical Education of Catalonia (INEFC), University of Barcelona (UB), Barcelona, Spain; ^2^Unió Esportiva Santboiana, División de Honor de Rugby, Sant Boi de Llobregat, Spain; ^3^Research Centre in Sports Sciences, Health Sciences and Human Development, CreativeLab Research Community, University of Trás-os-Montes and Alto Douro, Vila Real, Portugal; ^4^Futbol Club Barcelona, Barcelona, Spain; ^5^FC Internazionale Milano, Milan, Italy; ^6^Carnegie Applied Rugby Research Centre, Institute for Sport, Physical Activity and Leisure, Leeds Beckett University, Leeds, United Kingdom; ^7^Leeds Rhinos RLFC, Leeds, United Kingdom; ^8^Yorkshire Carnegie RUFC, Leeds, United Kingdom; ^9^England Performance Unit, The Rugby Football League, Leeds, United Kingdom; ^10^School of Science and Technology, University of New England, Armidale, NSW, Australia; ^11^Division of Exercise Science and Sports Medicine, Department of Human Biology, Faculty of Health Sciences, University of Cape Town, Sports Science Institute of South Africa, Cape Town, South Africa

**Keywords:** entropy, strength training, task constraints, team sports, adaptability

## Abstract

The aim of this study was to identify the changes in movement variability and movement velocity during a six-week training period using a resistance horizontal forward–backward task without (NOBALL) or with (BALL) the constraint of catching and throwing a rugby ball in the forward phase. Eleven elite male rugby union players (mean ± SD: age 25.5 ± 2.0 years, height 1.83 ± 0.06 m, body mass 95 ± 18 kg, rugby practice 14 ± 3 years) performed eight repetitions of NOBALL and BALL conditions once a week in a rotational flywheel device. Velocity was recorded by an attached rotary encoder while acceleration data were used to calculate sample entropy (SampEn), multiscale entropy, and the complexity index. SampEn showed no significant decrease for NOBALL (ES = -0.64 ± 1.02) and significant decrease for BALL (ES = -1.71 ± 1.16; *p* < 0.007) conditions. Additionally, movement velocity showed a significant increase for NOBALL (ES = 1.02 ± 1.05; *p* < 0.047) and significant increase for BALL (ES = 1.25 ± 1.08; *p* < 0.025) between weeks 1 and 6. The complexity index showed higher levels of complexity in the BALL condition, specifically in the first three weeks. Movement velocity and complex dynamics were adapted to the constraints of the task after a four-week training period. Entropy measures seem a promising processing signal technique to identify when these exercise tasks should be changed.

## Introduction

Resistance training is a key determinant of the physical conditioning process in elite rugby (Inness et al., 2016). It has been suggested that traditional resistance training tasks are too static and contradictory to the natural complex open system of team sports, which demands the self-organization of the large amount of degrees of freedom involved in the interaction between the environment and the dynamics of players’ decisions and actions (Travassos et al., 2011). Therefore, developing the ability to perform stable actions, i.e., the capacity to accelerate and decelerate (Seitz et al., 2015), under complex scenarios involving attuning interpersonal coordination (Duarte et al., 2012), as well as equipment and pitch space control in decision making (Greihaine et al., 2011), is very challenging but imperative at high levels of competition (Couceiro et al., 2013). In fact, rugby players need to be effective at sprinting while carrying a rugby ball (Pollard et al., 2018), which consequently increases the complexity of running, by altering the natural arm swing performed to counterbalance the hip rotation (Barr et al., 2015).

One of the most important variables to consider when designing an optimal resistance training program is the movement velocity (Bautista et al., 2016), so the training can be transferable to the tasks that require a developed capacity of body acceleration. However, the guidelines available in the current literature lack information on coordination patterns of the neuromuscular control system responses during training (England and Granata, 2007). By describing the effects emergent from different task constraints on such patterns, novel and important information about the players’ mechanisms of organic adaptation can be revealed (Mehdizadeh et al., 2015). Indeed, recent research identified motor variability as a key factor to describe the coordination features from the sensorimotor system operations and from the learning processes (Dhawale et al., 2017).

Recent research has found that the use of specific task constraints, such as carrying or passing a rugby ball during the execution of a functional eccentric overload resistance exercise, elicits different structures of variability in players’ body acceleration across multiple time scales, particularly toward higher level or systemic scales (Moras et al., 2018). One of the follow-up questions from this first body of evidence is related to the effect of time on the biological complexity responses in resistance training programs that use ball constraints, particularly associated to the acceleration outcomes and their effects on performance.

There are different approaches to analyze human movement and assess variability to identify changes in patterns and spatiotemporal characteristics (Stergiou et al., 2006; Preatoni et al., 2010, 2013; Dhawale et al., 2017; Moras et al., 2018). It has been recognized that linear measurements have several limitations, especially in determining movement degree of complexity and the time-dependent structure of a time series (Lipsitz and Goldberger, 1992). These limitations can be addressed by a non-linear approach, such as measures of entropy, to better describe healthy and pathological conditions (Costa et al., 2002), changes in postural control (Rhea et al., 2011; Lubetzky et al., 2018), assessment of running (Murray et al., 2017), tactical behavior in soccer (Gonçalves et al., 2017), or movement variability in resistance training tasks (Moras et al., 2018).

Entropy quantifies the amount of regularity and unpredictability of point-to-point fluctuations in large sets of time-series data (Richman and Moorman, 2000). Sample entropy (SampEn) and multiscale entropy (MSE) are two of the most popular methods for assessing data regularity in health and sports sciences (Busa and van Emmerik, 2016). Sample entropy measures the probability that similar sequences of points in a time-series remain similar within a tolerance level when a point is added to the sequence, in a single time scale (Richman and Moorman, 2000). On the other hand, MSE analysis has been suggested to be a better method to address the complexity inherent in the biological signals because it considers multiple spatial and temporal scales in a time series, reflecting the multiscale characteristics of the biological system operation (Costa et al., 2002, 2005; Gow et al., 2015). Particularly regarding movement variability, research is still limited to a few examples who suggest that it might be reduced as a function of practice (Newell and Vaillancourt, 2001; Wu et al., 2014) and experience (Ko and Newell, 2015; Williams et al., 2016). However, how movement variability decays over time during resistance training over the course of a training program, thus, how it affects players’ adaptive capacity, remains unclear. Therefore, the aim of this study was to identify the changes of movement variability, complexity index, and movement velocity with training in a resistance horizontal forward–backward task without (NOBALL) or with the constraint of catching and throwing a rugby ball in the forward phase (BALL) during a six-week training program.

It was hypothesized that movement variability and complexity index would decrease, and movement velocity would increase, over the course of a six-week training program, especially when using the constraint of catching and throwing a rugby ball. Conversely, the stabilization of movement variability, complexity index, and movement velocity can be used to identify an optimal moment to modify the task.

## Materials and Methods

### Participants

Eleven elite male rugby union players from a professional team in the Spanish league volunteered to participate in this study (mean ± SD: age 25.5 ± 2.0 years, height 1.83 ± 0.06 m, body mass 95 ± 18 kg, rugby practice 14 ± 3 years). All players were asked to avoid strenuous exercise during the study and informed about the procedures and possible risks while giving their informed consent before their admission. No players had any injuries through the study duration and the procedures complied with the Declaration of World Medical Association (2013) and were approved by the local ethics committee (21/20118/CEICEGC).

### Design

**FIGURE 1:**
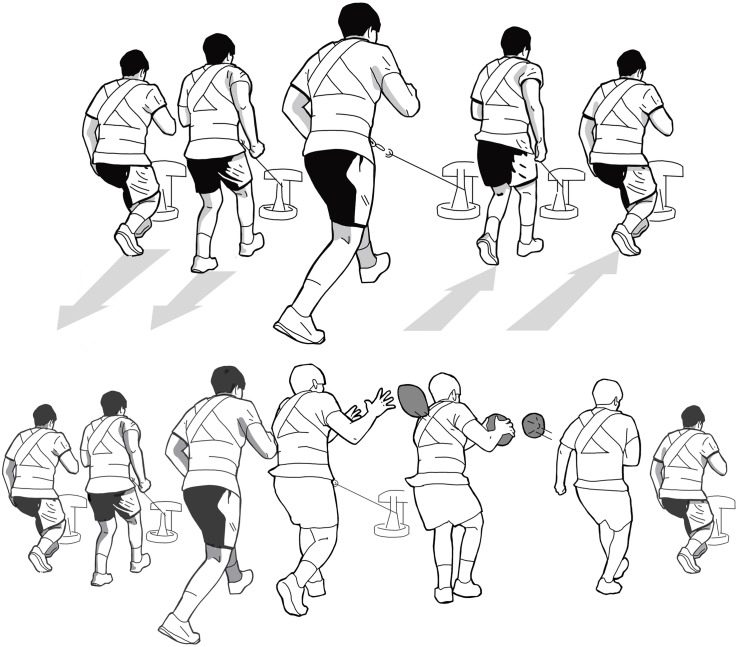
Horizontal movement with an inertial flywheel device with BALL **(below)** and NOBALL **(above)**.

The study was performed over 6 weeks. A recent meta-analysis about the effects of flywheel training on Strength-Related Variables show that the majority of these studies were carried out on periods of training between 5 and 10 weeks (Petré et al., 2018). However, more concretely, another recent study demonstrated that 4 weeks could be enough time to show muscle adaptation in flywheel resistance training (Illera-Domínguez et al., 2018). Further, in horizontal inertial flywheel training, which has more similarity to our study (de Hoyo et al., 2015; Gonzalo-Skok et al., 2016), differences in power and functional performance in 6- and 8-week period training were found. So, for these reasons, we hypothesize that 6 weeks could be enough time to find significant differences in both variables, movement velocity and movement variability. Since the players had no previous experience with this device prior to the experiment, participants underwent a familiarization session in which the horizontal movement with an inertial flywheel device was performed at a submaximal intensity in two conditions (BALL and NOBALL). When performing the BALL condition, an expert player made a pass from the right side two meters away. The participant caught the ball over the forward movement, synchronized with the first step (Figure 1). Then, during the second step, the participant passed the ball to another expert player standing 2 m away at the other side. Emphasis was placed on the importance of keeping the inertial flywheel rope tight. The training protocol was performed once a week during 6 weeks and included a warm-up, where the players performed 5 min of cycle ergometer, 5 min of general active mobility, two progressive sprints of 10 m, 10 movements at maximum speed forward and backward of 4 m, and five movements of maximum speed with changes of direction of 3 m. Afterward, the participants randomly performed eight repetitions of NOBALL and BALL with 3 min of rest between exercises. In the first two repetitions the intensity was progressively increased, while the last six were performed at maximal voluntary effort. During data collection, participants did not receive any verbal information on the quality of the movement performed or the outcomes of the test. Data collection took place during the competitive season.

### Procedures

The inertial flywheel device (Byomedic System SCP, Barcelona, Spain) consists of a metal flywheel (diameter: 0.42 m) with up to 16 weights (0.421 kg each weight), that can be added along the top edge of the flywheel perimeter. The device is comprised of a cone attached above a flywheel, and as the axle spins, a rope winds and unwinds around the cone. The concentric action unwinds the rope and the eccentric action occurs during rewinding. The force applied in the eccentric action to bring the flywheel to a stop will rely on the kinetic energy generated during the concentric action (Vicens-Bordas et al., 2018). To change the resistance to movement, the moment of inertia can be modified by adding any number of the 16 weights to the edge of the flywheel and also by selecting one of the four positions (P1, P2, P3, or P4) by changing the location of the pulley that is close to the cone. Position 1 and 16 weights were selected for this study, because these can generate the highest levels of mean force (Vázquez-Guerrero et al., 2016). The moment of inertia for the flywheel was 0.27 kg m^2^. Movement velocity was measure by a rotational encoder (Chronojump, Barcelona, Spain) which measures the spinning velocity of the axis of the flywheel device.

The participants’ acceleration performed in both conditions was measured using the inertial measurement unit WIMU (Realtrack Systems, Almeria, Spain), with processing capability consisting of a 3D accelerometer recording at 1000 Hz. The accelerometer was placed on an elastic waist belt close to the sacrum of each player. This position provided the best indication of whole body movement, as the location is close to the player’s center of mass (Montgomery et al., 2010).

Four repetitions of the NOBALL and BALL conditions were considered for further analysis. Each sample record contained 13879 ± 1900 data points for NOBALL and 14703 ± 1804 for BALL. In addition, the raw signal was obtained from the system specific software (WIMU Software, Realtrack Systems SL, Almería, Spain) to calculate total acceleration (at) based on the summation of vectors in three dimensions: mediolateral (*x*), anteroposterior (*y*) and vertical (*z*) (Moras et al., 2018). The mean velocity was recorded for the same four repetitions, registered with a rotary axis encoder, and analyzed with the software of chronojump (Chronojump, Barcelona, Spain).

The acceleration data were used to calculate entropy measures across a single time scale (SampEn) and across a range of time-scales (MSE), according to Chen et al. (2006) and Costa et al. (2002), using dedicated routines written in Matlab^®^ (The MathWorks, MA, United Sates). Also, the Complexity Index (Gow et al., 2015) was calculated as the area under each of the MSE curves to provide information on the integrated complexity of the system, over the time scales of interest (Busa and van Emmerik, 2016; Hansen et al., 2017). The mean velocity recorded from the encoder was also included in the analysis.

### Statistical Analysis

Data normality and homogeneity was assessed using Shapiro–Wilk and Levene tests, respectively. Data analyses were performed using PASW Statistics 21 (SPSS, Inc., Chicago, IL, United States). The level of statistical significance was set at *p* < 0.05. The response variable (SampEn, complexity index, and mean velocity) were analyzed using a repeated measure analysis of variance (ANOVA) to address the main and interactive effects between weeks, comparing the baseline (week 1) with all other weeks.

The comparisons were also assessed via standardized mean differences (Cohen’s d) and respective 90% confidence intervals. Thresholds for effect sizes statistics were <0.20, trivial; 0.20–0.59, small; 0.6–1.19, moderate; 1.20–1.99, large; and >2.0, very large (Hopkins et al., 2009). Movement velocity and Complexity Index values under BALL and NOBALL conditions were also adjusted to a third-degree polynomial for a better visualization of these variables in summarizing the effects of the six-week training protocol.

Finally, Bland–Altman analysis was used to assess biases of the variables (SampEn, complexity index and mean velocity) between conditions (Bland and Altman, 1995).

## Results

**FIGURE 2:**
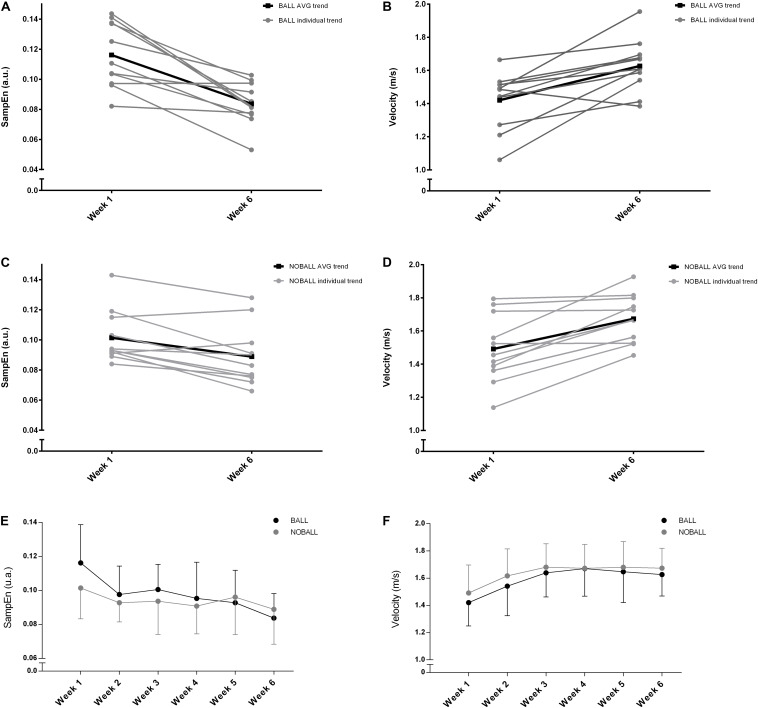
Individual trends, average, and standard deviation. SampEn and velocity in BALL **(A,B)** and NOBALL **(C,D)** compared between the baseline (week 1) and final week (week 6). Group average and standard deviation for SampEn **(E)** and velocity **(F)** in both conditions.

The individual trends, average, and standard deviation across the 6 weeks for SampEn and movement velocity in BALL and NOBALL conditions are shown in Figure 2. SampEn presented higher values in the first four weeks for BALL and in the last two weeks for NOBALL (Figures 2A,C,E). However, movement velocity presented higher values across the whole training period for NOBALL, although the values were similar in the last two weeks (Figures 2B,D,F).

**FIGURE 3:**
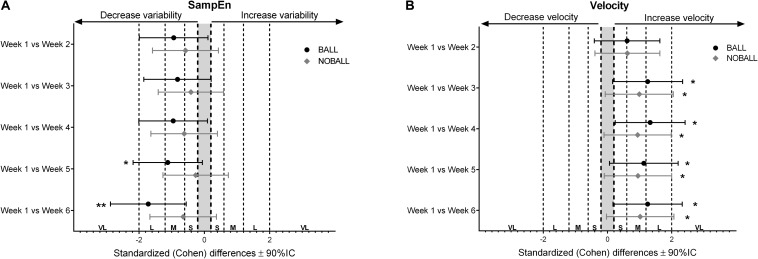
Standardized Cohen’s differences between the baseline (week 1) and the subsequent weeks for SampEn **(A)** and velocity **(B)** in both conditions. Error bars indicate uncertainty in true mean changes with 90% confidence intervals. Also, the significant differences were shown as *p < 0.05 and **p < 0.01. VL, very large; L, large; M, moderate; S, small.

When SampEn was compared between the baseline (week 1) and the subsequent weeks in the BALL condition, there were no significant changes, but there were moderate effects in the first four weeks, significant changes in the fifth week (*p* = 0.015) with moderate effects, and significant changes in the last week (*p* = 0.007) with a large effect (Figure 3A). By contrast, there were no significant differences in NOBALL conditions between weeks (Figure 3A). Also, when movement velocity was compared between the baseline (week 1) and the subsequent weeks in the BALL condition, there were significant changes in third (*p* = 0.010), fourth (*p* = 0.045), fifth (*p* = 0.029), and sixth (*p* = 0.047) weeks with moderate and large effects (Figure 3B). For the NOBALL condition there were significant changes in third (*p* = 0.012), fourth (*p* = 0.048), fifth (*p* = 0.027), and sixth (*p* = 0.025) weeks with moderate effects (Figure 3B).

**FIGURE 4:**
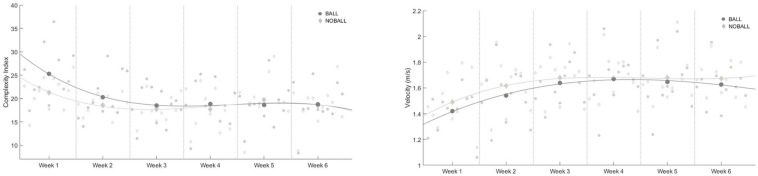
Complexity indexes and movement velocity values. BALL and NOBALL conditions adjusted to a third-degree polynomial.

When complexity indexes were compared between the baseline (week 1) and the subsequent weeks in the BALL condition, there were significant changes for every week (*p* ≤ 0.05), except with the fourth week. By contrast, there were no significant differences in NOBALL conditions between the training weeks. The results from the complexity index and movement velocity are presented in Figure 4, smoothed using a third-degree polynomial for a better visualization. There were higher levels of complexity in the BALL conditions, specifically in the first three weeks.

**FIGURE 5:**
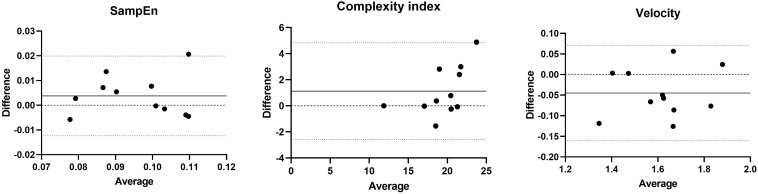
Bland and Altman plots, with the representation of the limits of agreement (dotted line) and bias (line).

Bland-Altman plots are presented in Figure 5. The resulting graph is a scatter plot *xy*, in which the *y* axis shows the difference between the conditions (BALL–NOBALL) and the *x* axis represents the average of these measures.

## Discussion

This study aimed to identify how movement variability and movement velocity changes during six weeks of training including a resistance horizontal forward–backward task without (NOBALL) or with the constraint of catching and throwing a rugby ball in the forward phase (BALL). In general, the results suggested that movement velocity and movement variability were adapted to the constraints after four weeks of training.

The baseline values at week 1 showed higher movement variability in the BALL when compared to NOBALL condition, supporting results recently reported (Moras et al., 2018). It was also possible to identify that movement variability remained higher until the fifth week of training, showing that using the ball as a constraint during this functional resistance training exercise demands higher levels of coordination patterns, stimulating the beneficial and adaptive aspects of variability in system function (van Emmerik and van Wegen, 2002).

The results also showed that movement variability across the six-week training period had a moderate and large reduction from week 1 to week 6 and a significant decrease in the weeks 5 and 6 for the BALL condition. This decrease might be due to an improved ability to control the coordination of the ball pass through practice (Ko and Newell, 2015; Williams et al., 2016). Based on the principle of optimality, sensory estimation could minimize uncertainty across optimal integration, and minimize variability in motor output through optimal control (Bays and Wolpert, 2007).

After four weeks of training, there was a stabilization on the BALL condition whichh was noted not only in a single temporal scale, as evidenced by SampEn values, but also when different temporal scales are considered, as seen in the complexity index results. The complexity index represents how systems are integrated from its lowest (organic) to highest (systemic) scale levels. When constraints are applied to resistance training, there seems to be changes in the system coordination patterns (Oliveira et al., 2013; Moras et al., 2018), however, the training process seems to regulate movement stability and adaptability (van Emmerik and van Wegen, 2002) to the point where the motor system is adapted to the environmental perturbations. The present study reports evidence that corroborates on the beneficial and adaptive aspects of variability during resistance training (van Emmerik and van Wegen, 2002) but, most importantly, reports details about the time-course of the effects related to the use of these different and unusual constraints. The results showed that four weeks were enough time for the task constraint to become too predictable for the players, therefore, not requiring substantial organic adaptations. Note that, after four weeks of resistance training, the complexity index was similar for both conditions, whereby the application of the constraints loses its effect and exercise tasks should be modified. Although the assessment of movement variability provides information about coordinative adaptations, during resistance training, the velocity at which a given load is displaced determines the strength and power adaptations at the muscular level (Bautista et al., 2016).

As expected in the NOBALL condition, the movement velocity output was higher than BALL, possibly due to the lower level of coordination required to perform the task. However, using match specific constraints during resistance training achieved more improvement between weeks. While NOBALL has a higher dependency on players’ capacity of improving force and velocity, BALL demands a higher level of motor skill, because it involves the coordination of carrying a ball while developing rapid accelerations. After three weeks of resistance training there were significant differences in the velocity for both conditions compared with week 1. Nevertheless, from week 4 to the end of the 6-week training period, the movement velocity did not change with training with or without the ball constraint. Thus, the result found in the current study suggests that velocity adaptations are reached before the movement variability, maybe because neuromuscular adaptations to human velocity and human variability are associated with different regulatory mechanisms (Hedayatpour and Falla, 2015). In team sports, the effectiveness of resistance training to improve sport performance depends upon the process of adaptations in terms of temporal structure changes (movement variability) and output performance magnitude (movement velocity). Therefore, the present study provides evidence that might better guide the training process, establishing optimal challenging points for resistance exercises and combining physical and coordinative tasks.

A previous study showed how entropy measures detect increased movement variability in resistance training when the ball is used like a constraint (Moras et al., 2018). The present study helps us to understand how the learning process inherent to a period of functional resistance training using a ball constraint changes the variability of the acceleration and affects performance across time. This study shows how entropy serves as an alternative tool to identify not only the changes in movement variability, but its time-course during a training period. This way, the trainers can structure the exercises to enhance players’ performance according to the field tasks and match demands required (McLaren et al., 2016) by efficiently combining physical and coordinative capacities in resistance training.

### Limitations

One of the main limitations of the current study was the low sample size (*n* = 11) and all of the players belonging to the same club. Nevertheless, these were expert players at the maximum level of competition in Spain. Rugby Union is a team sport with high levels of injury (Ball et al., 2017), especially at the maximum level (Yeomans et al., 2018), therefore, completing the training protocol during six weeks continuously in the competitive season period with enough healthy players was already an important milestone achieved.

## Conclusion

Six weeks of resistance training decreases movement variability and increases velocity, especially when catching and throwing a rugby ball. Despite that, the success in the application of tasks constraints might be compromised after four weeks of training. Coaching staffs can consider this moment as the key to decide whether to modify the task.

## Practical Implications

(1) Entropy measures can be used as a way of evaluating the ongoing appropriateness of an exercise stimulus to optimize adaptation. Entropy measures can be used by strength and conditioning coaches to identify when exercise tasks should be modified to trigger further adaptations.
(2) Entropy can help to identify the optimal challenge point, therefore maintaining movement variability and preventing a plateau in exercise adaptations. The use of the ball during a functional resistance training task will result in higher trainability, especially during the first four weeks. This is due to the increased complexity of the exercise.
(3) Strength and conditioning coaches should consider the inclusion of the ball when targeting the development of coordination within a periodized training program.

## Data Availability Statement

The datasets generated for this study are available on request to the corresponding author.

## Ethics Statement

The studies involving human participants were reviewed and approved by Catalan Sports Council (Generalitat de Catalunya) ethics committee (21/20118/CEICEGC). The patients/participants provided their written informed consent to participate in this study.

## Author Contributions

BF-V and GM conceived and designed the experiments. BF-V, GM, and JG performed the experiments. BF-V, GM, JS, JE, and JG analyzed the data. BF-V wrote the first draft of the manuscript. BF-V, GM, JS, JE, JT-F, and BJ wrote, reviewed and edited the manuscript. All authors read and approved the submitted version of the manuscript.

## Conflict of Interest

The authors declare that the research was conducted in the absence of any commercial or financial relationships that could be construed as a potential conflict of interest.
